# Identification of GGC Repeat Expansions in *ZFHX3* among Chilean Movement Disorder Patients

**DOI:** 10.1002/mds.30242

**Published:** 2025-06-03

**Authors:** Paula Saffie‐Awad, Abraham Moller, Kensuke Daida, Pilar Alvarez Jerez, Zhongbo Chen, Zachary B. Anderson, Mariam Isayan, Kimberly Paquette, Sophia B. Gibson, Madison Fulcher, Abigail Miano‐Burkhardt, Laksh Malik, Breeana Baker, Paige Jarreau, Henry Houlden, Mina Ryten, Bida Gu, Mark J.P. Chaisson, Danny E. Miller, Pedro Chaná‐Cuevas, Cornelis Blauwendraat, Andrew B. Singleton, Kimberley J. Billingsley

**Affiliations:** ^1^ Clínica Santa María Santiago Chile; ^2^ Center for Alzheimer's and Related Dementias, National Institute on Aging and National Institute of Neurological Disorders and Stroke, National Institutes of Health Bethesda Maryland USA; ^3^ Laboratory of Neurogenetics, National Institute on Aging, National Institutes of Health Bethesda Maryland USA; ^4^ Department of Neurodegenerative Disease UCL Queen Square Institute of Neurology, University College London London UK; ^5^ Department of Genetics and Genomic Medicine Great Ormond Street Institute of Child Health, University College London London UK; ^6^ NIHR Great Ormond Street Hospital Biomedical Research Centre University College London London UK; ^7^ Division of Genetic Medicine, Department of Pediatrics University of Washington Seattle Washington USA; ^8^ Department of Neurology and Neurosurgery National Institute of Health Yerevan Armenia; ^9^ Department of Genome Sciences University of Washington Seattle Washington USA; ^10^ Department of Neuromuscular Disease Queen Square Institute of Neurology, UCL London UK; ^11^ Department of Genetics and Genomic Medicine Great Ormond Street Institute of Child Health, UCL London UK; ^12^ NIHR Great Ormond Street Hospital Biomedical Research Centre UCL London UK; ^13^ UK Dementia Research Institute University of Cambridge Cambridge UK; ^14^ Department of Clinical Neurosciences, School of Clinical Medicine University of Cambridge Cambridge UK; ^15^ Department of Quantitative and Computational Biology University of Southern California Los Angeles California USA; ^16^ Department of Laboratory Medicine and Pathology University of Washington Seattle Washington USA; ^17^ Brotman Baty Institute for Precision Medicine University of Washington Seattle Washington USA; ^18^ Centro de Trastornos del Movimiento, Facultad de Ciencias M ´edicas Universidad de Santiago de Chile Santiago Chile

**Keywords:** Latin American Population, Long Read Sequencing, Spinocerebellar Ataxias, Tandem Repeat Expansions, ZFHX3 gene

## Abstract

**Background:**

Hereditary ataxias are genetically diverse, yet up to 75% remain undiagnosed due to technological and financial barriers. The GGC repeat expansion in *ZFHX3*, responsible for spinocerebellar ataxia type 4 (SCA4), has only been described in individuals of Northern Europeandescent.

**Objective:**

Uncover the genetic etiology of suspected hereditary movement disorders.

**Methods:**

We performed Oxford Nanopore long‐read genome sequencing on 15 individuals with suspected hereditary movement disorders. Using variant calling and ancestry inference tools.

**Results:**

We identified *ZFHX3* GGC expansions (47–55 repeats) in 4 patients with progressive ataxia, polyneuropathy, and vermis atrophy. One presented with rapidly progressive parkinsonism–ataxia, expanding the known phenotype. Longer expansions correlated with earlier onset and severity. All carriers shared single nucleotide variants (SNVs) associated with the Swedish founder haplotype, and methylation analysis confirmed allele‐specific hypermethylation.

**Conclusion:**

These represent the first SCA4 cases identified outside Northern Europe. Our findings highlight the value of long‐read sequencing in resolving undiagnosed movement disorders. Published 2025. This article is a U.S. Government work and is in the public domain in the USA. *Movement Disorders* published by Wiley Periodicals LLC on behalf of International Parkinson and Movement Disorder Society.

In Hereditary ataxias are clinically and genetically heterogeneous, resulting from a wide range of genetic variant types, including short tandem repeats (STRs), single nucleotide variants (SNVs), and structural variants (SVs) such as large insertions, deletions, and other complex rearrangements. Molecular diagnosis remains challenging, with a diagnostic yield around 75%^1^ particularly in underrepresented populations, due to limitations in technology and access to comprehensive testing. In Chile, a diagnostic yield of only 23% has been reported for ataxias.^2^

SCA4 was first mapped to chromosome 16q22.1 over 25 years ago in a large Utah‐based family of Swedish ancestry.^3^ Its underlying genetic cause was recently identified, as an uninterrupted GGC repeat expansion in the final exon of *ZFHX3* gene, possibly linked to a single founder Northern European haplotype.^4^, ^5^ Notably, this expansion has not been identified in non‐European population^6^, ^7^ despite screening in Japanese^8^ and Brazilian cohorts.^9^ Expanded alleles typically range from 42 to 74 repeats, whereas normal alleles are generally shorter (14–31 repeats) and often contain interruptions by other sequences. Alleles in the 32–41 range have uncertain clinical significance.^10^ Clinically, SCA4 is characterized by a combination of cerebellar ataxia and sensory axonal neuropathy, frequently accompanied by dysautonomia and oculomotor abnormalities.^11^ A notable anticipation phenomenon correlated with repeat size, with a typical age of onset between 30 and 50 years.^4^, ^5^, ^6^

In this study, we performed Oxford Nanopore Technologies (ONT) genome long‐read sequencing on 15 individuals with suspected hereditary movement disorders, including ataxia and parkinsonism. This cohort was selected to maximize the diagnostic yield in genetically unresolved cases where repeat expansions or structural variants were suspected. We report the identification of *ZFHX3* expansions in 4 patients and describe the clinical, genetic, and epigenetic features of these cases. Here, we present these findings and emphasize the advantages of long‐read sequencing for accurately diagnosing SCA4 and other neurodegenerative diseases.

## Patients and Methods

### Patients and Participants

A total of 15 samples were collected from individuals with suspected hereditary movement disorders, ataxia, ataxia‐parkinsonism, parkinsonism, or atypical parkinsonism (Table S1). For this pilot study, we selected patients who were most likely to carry an undetected pathogenic variant, including those with negative results from prior exome and/or repeat expansion testing. All participants provided written informed consent, and the study was approved by the Chilean ethics committee.

### DNA Extraction and Sequencing

DNA was extracted from frozen blood in Chile using a publicly available protocol (https://www.protocols.io/view/protocol‐purification‐of‐dna‐from‐whole‐blood‐usin‐c7ypzpvn, DOI: 10.17504/protocols.io.n92ldmx3ol5b/v1),^12^ and sequencing was performed using PromethION sequencing ONT (*MinKNOW*, version 24.02.10). Each sample yielded >115 GB of data (Table S2).

### Variant Calling and Ancestry Analysis

**FIG. 1:**
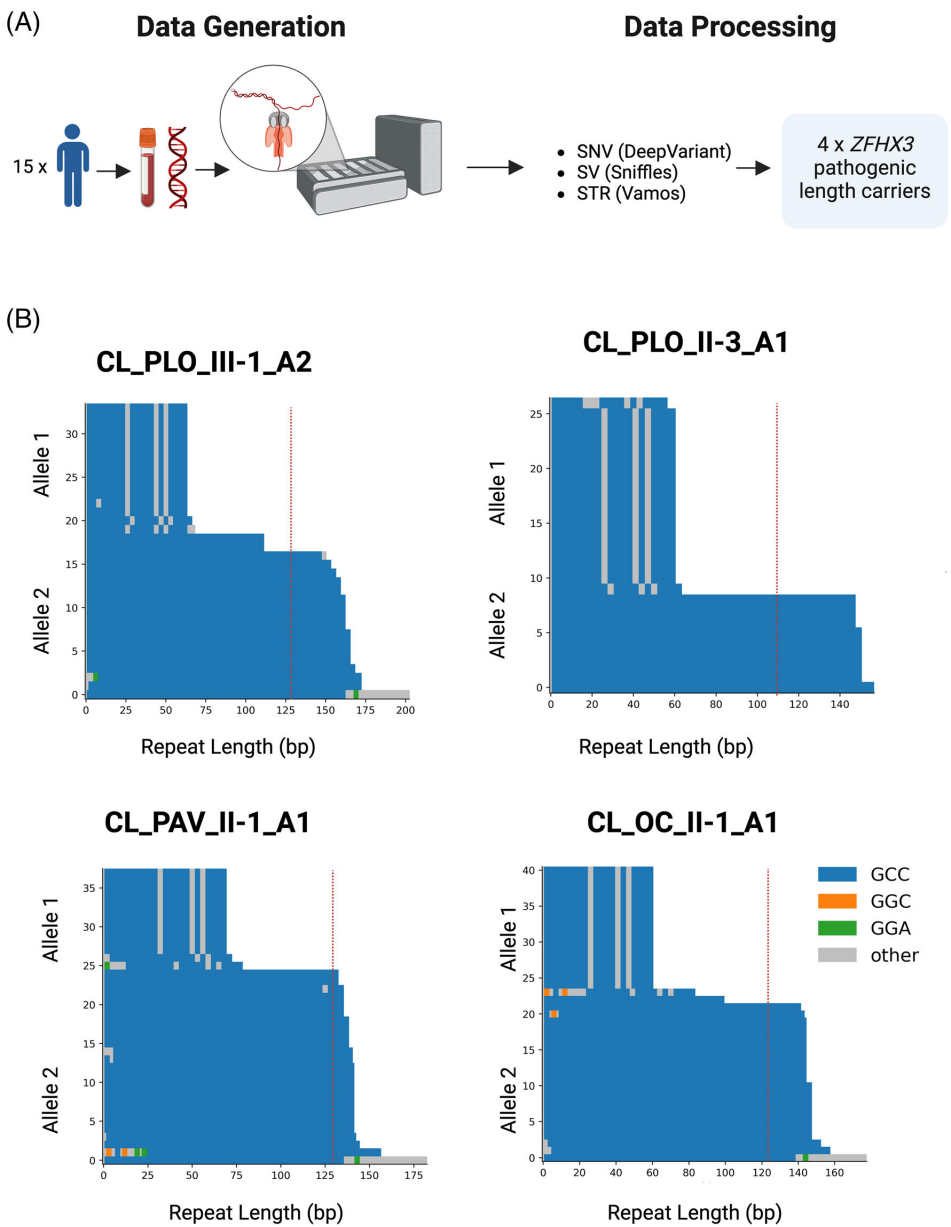
(**A**) Schematic overview of the study design. (**B**) Waterfall plots displaying Oxford Nanopore Technologies (ONT) long‐read sequencing data for the four predicted *ZFHX3* GGC repeat carriers. The red dotted line marks the pathogenic threshold. Created using BioRender.com.

Basecalling was conducted with *Dorado* v0.7.1, and reads were aligned to GRCh38 using *Minimap2* v2.28. Variant calling included SNVs (identified with *PEPPER‐Margin‐DeepVariant* 0.8^13^), SVs (*Sniffles* 2.4^14^), and STRs (*Vamos* 2.1.3^15^). A detailed overview of the workflow used for these analyses is presented in Figure 1A. Ancestry was inferred using GenoTools with reference from the 1000 Genomes Project, Human Genome Diversity Project, and Ashkenazi Jewish dataset.^16^, ^17^

### Haplotype Analysis

To compare haplotypes, we took the SNV calls for our samples and looked for the six ultra‐rare SNVs coming from the Swedish ancestry founder effect as reported in Chen et al.^18^ We then plotted the haplotypes for visual comparison using the following script: https://github.com/zanderson82/SNP‐Haplotype‐Plotting/tree/main.

### Methylation Analysis

Methylation profiles were generated using *modbamtools* v0.4.8.^19^ Haplotagged BAM files from *PEPPER‐Margin‐DeepVariant* and *Gencode* v38 GRCh38 gene tracks were used as input. The —hap option was used to display haplotype‐specific methylation frequency.

### Control Dataset Repeat Size Estimation

We analyzed 438 control samples from three long‐read control cohorts; the North American Brain Expression Consortium (NABEC; n = 205, dbGaP Accession phs001300.v4.p1), the Human Brain Collection Core^20^ (HBCC; n = 133), and 100 individuals of mixed ancestry from the 1000 Genomes^21^ (1000G) Long‐Read Project, using the same STR caller (*Vamos*) to ensure methodological consistency with the Chilean samples.

### Code Availability

All scripts used for data processing and analysis are available at https://github.com/molleraj/CARDlongread-chile-data-processing.

## Results

### Clinical Features of Affected Individuals

The clinical features of all 4 individuals are summarized in Table 1. All patients initially presented with progressive cerebellar ataxia and sensory polyneuropathy. Two affected individuals (CL_PLO_III‐1_A2 and CL_PLO_II‐3_A1) belonged to the same family. CL_PLO_III‐1_A2, a 32‐year‐old woman carrying 55 GGC repeats, had a 10‐year disease duration with mild ataxia (SARA 10), EMG‐confirmed polyneuropathy, and chronic cough. She exhibited no dysarthria, motor neuron signs, or cognitive impairment. Magnetic resonance imaging (MRI) revealed mild cerebellar atrophy. Her older relative, CL_PLO_II‐3_A1 (66 years, 49 GGC repeats), presented with severe ataxia (SARA 28), spasticity, a positive Babinski sign, polyneuropathy with hypopalesthesia, dysarthria, mild cognitive decline (MoCA 21), bladder dysfunction, and rapid eye movement (REM) sleep behavior disorder. MRI showed cerebellar vermis atrophy. The two unrelated individuals included CL_PAV_II‐1_A1, a 60‐year‐old woman with 47 GGC repeats and an 8‐year disease duration, who exhibited moderate gait ataxia (SARA 19), confirmed polyneuropathy (Fig. S4Ia), dysarthria, chronic cough, and upper spinal cord changes on MRI (Fig. S1Ib,c). No cognitive decline was observed, and there was no reported family history of neurological disorders. The fourth case, CL_OC_II‐1_A1, a 44‐year‐old man with 49 GGC repeats, had a 5‐year history of a rapidly progressive ataxia‐parkinsonism phenotype with severe ataxia, spasticity, a positive Babinski sign, myoclonus, EMG‐confirmed moderate‐to‐severe polyneuropathy (Fig. S1IIa), cognitive decline, bladder incontinence, constipation, and gaze palsy. MRI revealed marked cerebellar atrophy (Fig. S1IIb,c; see Video 1).

**TABLE 1:** Clinical characteristics of the four *ZFHX3* GGC repeat carriers

Category	CL_PLO_III‐2_A2	CL_PLO_II‐3_A1	CL_PAV_II‐1_A1	CL_OC_II‐1_A1
Length longest allele (pathogenic cut off = 42)	55	49	47	49
Gender	Female	Male	Female	Male
Age	42	75	68	44
Age at onset (AAO)	32	66	60	39
Disease duration	10	9	8	5
First degree family History	Yes	Yes	No	Yes
Ataxia	Yes	Yes	Yes	Yes
Dysarthria	No	Yes	Yes	Yes
SARA Scale	10.0	28.0	19.0	NA
Upper motor neuron involvement	No	Spasticity, Babinski sign	No	Spasticity, Babinski sign
Sensitive polyneuropathy	Confirmed with EMG	Clinically assessed	Confirmed with EMG	Confirmed with EMG
Dysautonomia	No	Yes, Bladder incontinence	No	Severe bladder incontinence and constipation
Cognitive decline	No	Mild	No	Moderate
Other symptoms	Chronic cough	RBD	Chronic cough	PKN, gaze paralysis, myoclonus, severe neuropathic pain
MRI findings	Mild cerebellar atrophy	Vermis cerebellar atrophy	Upper vermis atrophy and upper spinal cord	Cerebellar atrophy
Previous study	Negative repeat expansion panel and exome sequencing	Negative repeat expansion panel and exome sequencing	No	Negative repeat expansion panel
Vertigo	No	No	No	No
Medical history	Asthma	Asthma	High blood pressure, arthrosis	Migraine

Abbreviations: SARA, Scale for the Assessment and Rating of Ataxia; EMG, electromyography; PKN, for parkinsonism; RBD, rapid eye movement sleep behavior disorder; MRI, magnetic resonance imaging.

**Video 1:** This video demonstrates key clinical features in a 44‐year‐old man with a *ZFHX3* GGC repeat expansion. The first segment shows a severe gait disturbance, requiring assistance, with a wide‐based gait and instability. The second segment highlights complex ophthalmoplegia. The final segment illustrates the ataxia‐spasticity spectrum. [Color figure can be viewed at wileyonlinelibrary.com]

### Genetic Profiling

Genetic ancestry inference using GenoTools confirmed that all 15 Chilean participants clustered with the Latino/Admixed American (AMR) population (Fig. S2). Among the cohort, we identified 4 patients from three independent families, carrying pathogenic‐length *ZFHX3* GGC repeat expansions ranging from 47 to 55 units (Fig. 1b). No other pathogenic‐length STR expansions were detected (Table S3), nor known pathogenic SNVs or SVs were identified in these individuals. The lengths of *ZFHX3* GGC repeats in the control cohorts varied from 11 to 29 units (Fig. S3). No control sample from any cohort demonstrated a repeat expansion ≥42 units.

**FIG. 2:**
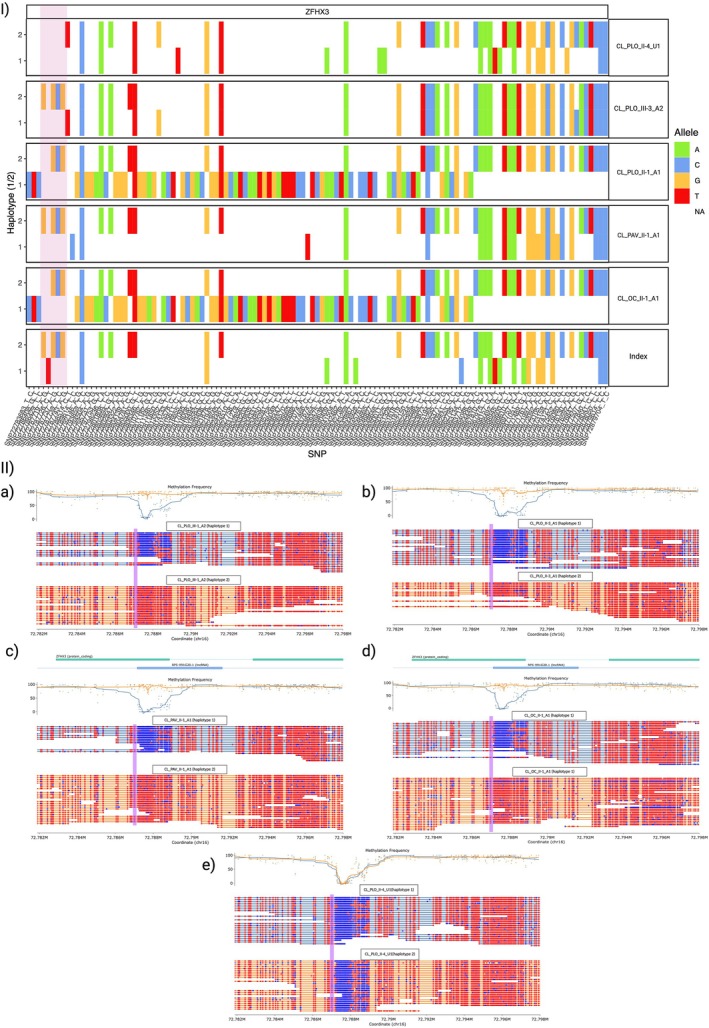
(I) Haplotype analysis of *ZFHX3* GGC repeat expansion carriers. Out of the six rare single nucleotide variants (SNVs) reported as part of the distance common founder event, four were found in our samples within the repeat region (highlighted by the pink box) and compared to the Utah index patient from Chen et al. ^18^ These SNVs are missing in the unaffected Chilean individual. (II) Haplotype‐specific differential methylation around the expansion. (a–d) Modbamtools plots of methylation frequency for the four heterozygous expansion carriers. Methylation frequency is plotted at the top where haplotype 1 (blue) represents the non‐expanded allele and haplotype 2 (orange) represents the expanded allele. The *ZFHX3* gene track is overlaid at the top, showing the last two exons. Haplotype‐specific reads are shown at the bottom with blue sections of the reads denoting hypomethylation and red sections of the reads denoting hypermethylation. Purple box indicates repeat region. For all four carriers, the expanded allele is hypermethylated compared to the non‐expanded allele. (e) Modbamtools plot of an unaffected related individual, homozygous non‐repeat carrier, showing that hypomethylation in this region is expected under normal conditions.

An inverse relationship was observed between repeat length and age at onset (Fig. S4), with longer expansions associated with earlier clinical manifestation, although the trend did not reach statistical significance (*R* ^2^ = 0.54, *P* = 0.26) it aligns with previous reports. ^10^ Phased SNVs surrounding the repeat expansion revealed four of the six ultra‐rare SNVs previously described as part of the Swedish founder haplotype by Figueroa et al ^4^ and Chen et al. ^18^ These variants were present in all four carriers and absent in non‐carriers (Fig. 2I; Table S4).

Methylation profiling using haplotype‐resolved long‐read data demonstrated consistent hypermethylation around the expanded allele, specifically overlapping the final exon of *ZFHX3* (Fig. 2IIa–d). In contrast, the non‐expanded allele and samples from non‐carriers showed hypomethylation in the same region (Fig. 2IIe). This differential methylation pattern is consistent with prior observations and suggests a potential regulatory effect of the expansion on gene expression.

## Discussion

This study provides the first report of *ZFHX3* GGC repeat expansions in Latin America, expanding the known geographic and ancestral distribution of SCA4. It also represents the largest analysis of the *ZFHX3* STR to date using long‐read sequencing across diverse ancestries, incorporating 438 control samples from NABEC, HBCC, and the 1000 Genomes Project. These data support the previously proposed pathogenic threshold of ≥42 repeats.^5^ Our findings reinforce the presence of a Northern European founder haplotype, likely introduced via historical migration. Despite the admixed ancestry of the Chilean individuals, all carriers shared ultra‐rare SNVs characteristic of the Swedish founder background.

We observed intergenerational repeat instability in one family, with an expansion from 49 to 55 repeats, confirming the phenomenon of genetic anticipation and repeat‐length, associated earlier onset, as previously described.^10^ Although clinical severity could not be directly compared, our findings support the molecular evidence of anticipation. All affected individuals exhibited typical SCA4 features:^22^ ataxia, sensory neuropathy, and dysautonomia, whereas one case presented with parkinsonism. Although not previously reported in SCA4, parkinsonian features have been described in other SCAs, such as SCA2 and SCA3,^23^, ^24^ and may reflect broader involvement of basal ganglia circuits in SCA4.^25^ Further functional and neuroimaging studies are warranted to better characterize this expanded phenotype.

At the molecular level, we confirmed allele‐specific hypermethylation surrounding the expanded *ZFHX3* repeat, suggesting an epigenetic contribution to disease pathogenesis. Similar methylation changes are observed in other repeat expansion disorders, including *C9orf72*‐related ALS/FTD^26^, ^27^ and Fragile X syndrome,^28^ where they have been linked to transcriptional dysregulation. Additional studies are needed to explore how the *ZFHX3* expansion alters gene expression or splicing. Consistent with previous findings, the haplotype identified in our Chilean cases traces back to a common Swedish founder estimated to have originated approximately 2200 years ago. Its detection outside of Northern Europe highlights the need to consider founder alleles in admixed populations and suggests a wider historical dispersal of the pathogenic expansion.^18^

Despite these insights, several limitations remain. The small sample size limits generalizability, and the lack of available genomic data from South American populations restricts comprehensive assessment of *ZFHX3* variability. Expanding sequencing efforts in underrepresented regions is essential to better define the prevalence, phenotypic spectrum, and clinical implications of *ZFHX3* expansions. Although long‐read sequencing is well suited for identifying repeat expansions and SVs, it remains resource‐intensive. Adaptive sampling^7^ approaches may offer a more scalable solution, enabling efficient enrichment of ataxia‐associated loci for comprehensive variant detection, while reducing sequencing costs. Although orthogonal testing was not performed, the combined evidence from methylation profiling, haplotype analysis, and clinical correlation supports the validity of our findings. Moreover, ONT has been used in previous *ZFHX3* studies to resolve GGC repeat expansions and haplotype structure without orthogonal confirmation, reinforcing the confidence in our approach.^5^, ^6^, ^18^

In summary, this study confirms the presence of a *ZFHX3* GGC repeat expansion in Latin America, documents repeat instability and founder haplotype conservation, and broadens the clinical spectrum of SCA4 to include parkinsonism. Further efforts to improve access to comprehensive genetic diagnostics and to expand representation in sequencing cohorts will be critical to advance understanding and care for individuals with rare ataxias.

## Author Roles

(1) Research Project: A. Conception, B. Organization, C. Execution; (2) Statistical Analysis: A. Design, B. Execution, C. Review and Critique; (3) Manuscript Preparation: A. Writing of the First Draft, B. Review and Critique.

P.S.A.: 1A, 1C, 2C, 3A, 3B

A.M.: 1A, 1C, 2C, 3B

K.D.: 1A, 1C, 2C, 3B

P.A.J.: 1A, 1C, 2C, 3B

Z.C.: 1A, 1C, 2C, 3B

Z.B.A.: 1C, 2B, 3B

M.I.: 1C, 2B, 3B

K.P.: 1C, 2B, 3B

S.B.G.: 2C, 3B

M.F.: 1C, 2B, 3B

A.M.B.: 1C, 2B, 3B

L.M.: 1C, 2B, 3B

B.B.: 1C, 2B, 3B

P.J.: 2C, 3B

H.H.: 2C, 3B

M.R.: 2C, 3B

B.G.: 2C, 3B

M.J.P.C.: 2C, 3B

D.E.M.: 1C, 2B, 3B

P.C.C.: 1B, 1C, 3B

C.B.: 1A, 1C, 2C, 3B

A.B.S.: 1C, 2B, 3B

K.J.B.: 1A, 1B, 1C, 2A, 2B, 2C, 3A, 3B

## Full financial disclosure of all authors (for the preceding 12 months)

This work was supported in part by the Intramural Research Program of the National Institute on Aging (NIA) and the Center for Alzheimer's and Related Dementias (CARD), within the Intramural Research Program of the NIA and the National Institute of Neurological Disorders and Stroke (ZIANS003154, ZIAAG000538, AG000538), National Institutes of Health (NIH). Computational resources were provided by the NIH HPC Biowulf cluster (https://hpc.nih.gov). The authors declare no financial or non‐financial conflicts of interest related to this manuscript.

Individuals affiliated with CARD or the Laboratory of Neurogenetics at NIH are marked with an asterisk (*) below, as this is their funding source. None of the authors have received consultancies or honoraria, hold intellectual property rights, or receive royalties.

- P.S‐A.—Employment: Clínica Santa María, Santiago, Chile. Grant: Michael J. Fox Foundation. Advisory Boards: Biogen.
- P.A.J.*—Employment: Laboratory of Neurogenetics, National Institute on Aging, NIH, Bethesda, MD, USA; Department of Neurodegenerative Disease, UCL Queen Square Institute of Neurology, University College London, UK.
- C.B., A.B.S.*—Employment: Center for Alzheimer's and Related Dementias, National Institute on Aging and National Institute of Neurological Disorders and Stroke, NIH, Bethesda, MD, USA; Laboratory of Neurogenetics, National Institute on Aging, NIH, Bethesda, MD, USA.
- A.M.*, K.P.*, L.M.*, B.B.*, P.J.*, K.J.B.*—Employment: Center for Alzheimer's and Related Dementias, NIH, USA.
- K.D.*, M.F.*, A.M.‐B.*—Employment: Laboratory of Neurogenetics, NIH, Bethesda, MD, USA.
- Z.C.—Employment: UCL Queen Square Institute of Neurology, UK; Great Ormond Street Institute of Child Health, UCL, UK.
- Z.B.A.—Employment: University of Washington, Seattle, WA, USA.
- S.B.G.—Employment: Division of Genetic Medicine, Department of Pediatrics, University of Washington, Seattle, WA, USA; Department of Genome Sciences, University of Washington. Grant Support: NIH grant 5T32HG000035–29.
- D.E.M.—Employment: Division of Genetic Medicine, Department of Pediatrics, University of Washington, Seattle, WA, USA; Department of Laboratory Medicine and Pathology and the Brotman Baty Institute for Precision Medicine, University of Washington. Stock Ownership: MyOme. Advisory Boards: Oxford Nanopore Technologies (ONT), Basis Genetics. Partnerships: ONT, Pacific Biosciences, Basis Genetics. Grant Support: NIH DP5OD033357.
- M.I.—Employment: National Institute of Health, Yerevan, Armenia.
- H.H.—Employment: UCL Queen Square Institute of Neurology, UK. Grants: Michael J. Fox Foundation, MRC, Wellcome Trust, NIHR UCL/UCLH BRC.
- M.R.—Employment: UCL, UK; University of Cambridge, UK.
- P.C‐C: Employment: Universidad de Santiago de Chile, Centro de Estudios de Trastornos del Movimiento (CETRAM), Santiago, Chile.
- B.G., M.J.P.C.—Employment: University of Southern California, USA. Grant Support: NIH R01HG011649.

## Supporting information


**Figure S1. Panel Ia and IIa:** Electromyography (EMG) and nerve conduction studies of CL_PAV_II‐1_A1 (Ia) and CL_OC_II‐1_A1 (IIa) reflecting sensory neuropathy. **Panel Ib and IIb:** T1‐weighted MRI images showing upper cerebellar (vermal) atrophy in CL_PAV_II‐1_A1 (Ib) and CL_OC_II‐1_A1 (IIb), as well as upper cervical cord atrophy in Ib.

**Figure S2.** Scatter plot of principal components 1 and 2 from the Ancestry analysis Cluster plot of PC1 and PC2 of the ancestry analysis showed the Chilean samples clustering close to Latino/admixed American (AMR). AAC; African American/Afro‐Caribbean, AFR; African, AJ; Ashkenazi Jewish, AMR; Admixed American, CAS; Central Asian, EAS; Eastern Asian, EUR; European, FIN; Finnish, MDE; Middle Eastern, SAS; South Asian.

**Figure S3.** Control Dataset Repeat Size Estimation. (a) Distribution of ZFHX3 GGC repeat lengths in the Chilean samples (n = 15), read indicates pathogenic length carrier. (b) Distribution of ZFHX3 GGC repeat lengths in the 1000G control cohort (n = 100) comprising individuals of mixed ancestry. (c) Distribution of ZFHX3 GGC repeat lengths in the NABEC/HBCC control cohort (n = 338) comprising individuals of European and African‐admixed ancestry. The red dotted line marks the pathogenic threshold.

**Figure S4.** Inverse Correlation Between *ZFHX3* GGC Repeat Length and Age at Onset in SCA4 Patients. A negative trend is observed, but it is not statistically significant (*R* ^2^ = 0.54, *P* = 0.26).

**Table S1.** Clinical phenotypes of the individuals studied. Sample ID based on their country (CL for Chile), family ID, generation within the pedigree (Roman numerals: I, II, III, etc.), individual order within that generation (numbered from left to right in the pedigree), and affected status (A for affected, U for unaffected).
**Table S2.** Overview of long‐read sequencing data for the studied. N50 represents the read length at which 50% of total bases are in reads of that length or longer. Yield (Gb) indicates total bases sequenced. Mean coverage refers to the average sequencing depth.
**Table S3.** Overview of lengths per allele of known pathogenic expansion loci. The table includes chromosome (#chr) position, affected gene, associated disease, pathogenic repeat number, and repeat lengths for each individual.
**Table S4.** Summary results of haplotype analysis of six ultra‐rare SNVs associated with the Swedish ancestry founder effect.^4^, ^18^ Genotypes for the *ZFHX3* carriers at six SNV on chromosome 16 (hg38). Shared haplotypes are highlighted. Abbreviations: SNV, single nucleotide variant; hg38, human genome assembly GRCh38.

## Data Availability

The data that support the findings of this study are available from the corresponding author upon reasonable request.
