# Supplementary figures and images for: Identification of GGC Repeat Expansions in ZFHX3 among Chilean Movement Disorder Patients
Source: Mov Disord. 2025 Jun 3;40(7):1433–41. doi: 10.1002/mds.30242 (PMC12273613; doi:10.1002/mds.30242)

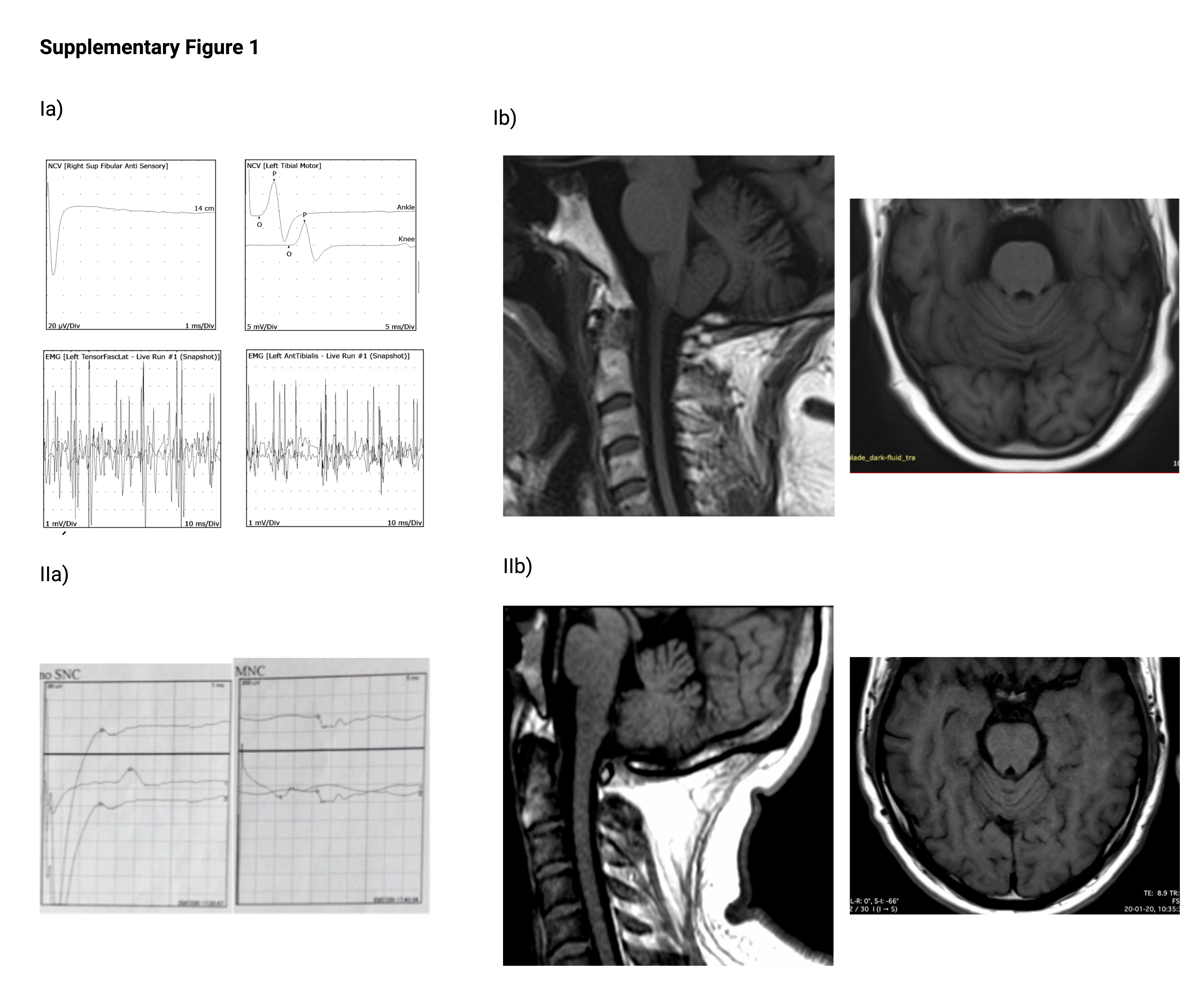

Supplement: Supplementary file 1 — Figure S1. Panel Ia and IIa: Electromyography (EMG) and nerve conduction studies of CL_PAV_II‐1_A1 (Ia) and CL_OC_II‐1_A1 (IIa) reflecting sensory neuropathy. Panel Ib and IIb: T1‐weighted MRI images showing upper cerebellar (vermal) atrophy in CL_PAV_II‐1_A1 (Ib) and CL_OC_II‐1_A1 (IIb), as well as upper cervical cord atrophy in Ib. [file MDS-40-1433-s002.jpg]

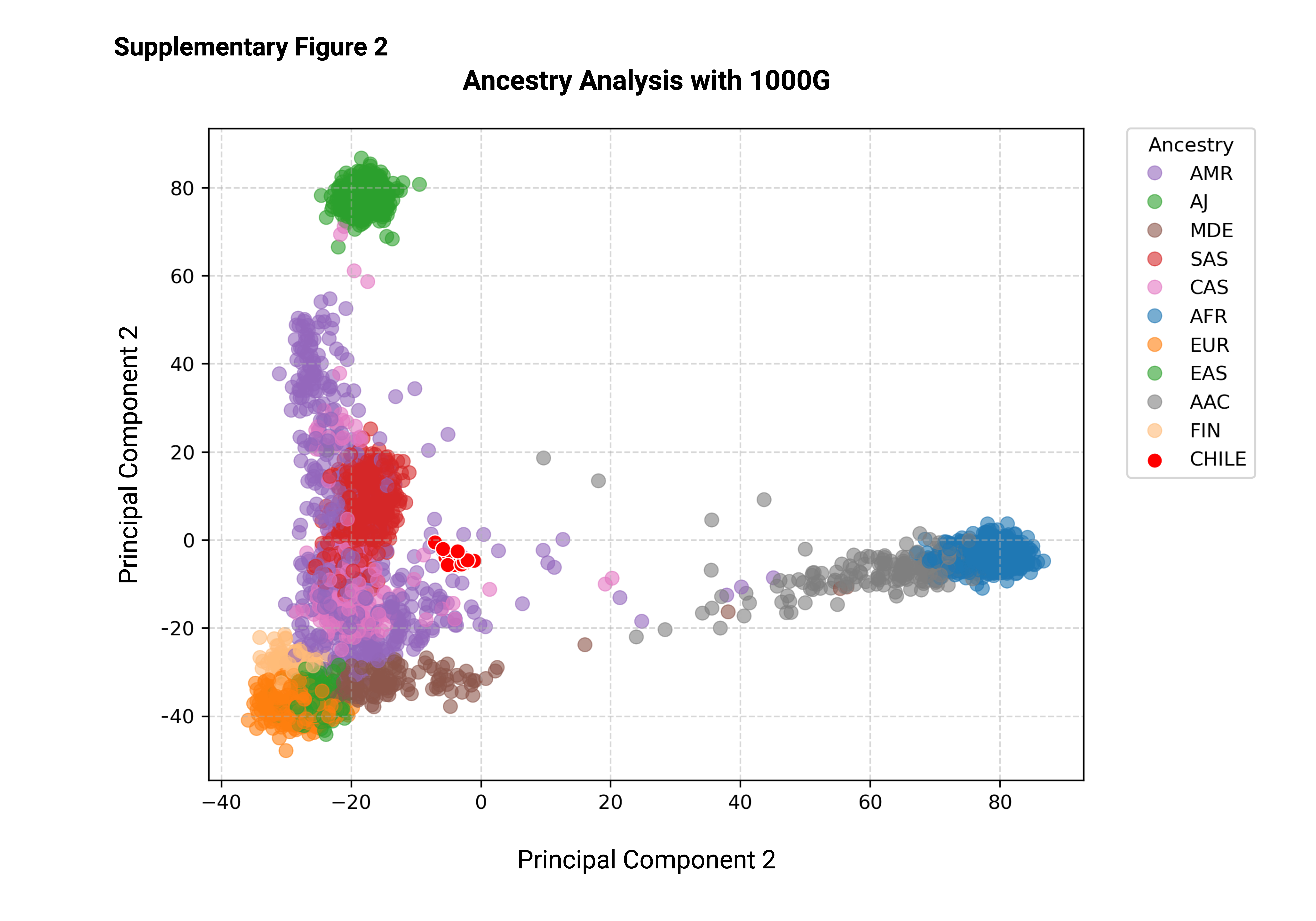

Supplement: Supplementary file 2 — Figure S2. Scatter plot of principal components 1 and 2 from the Ancestry analysis Cluster plot of PC1 and PC2 of the ancestry analysis showed the Chilean samples clustering close to Latino/admixed American (AMR). AAC; African American/Afro‐Caribbean, AFR; African, AJ; Ashkenazi Jewish, AMR; Admixed American, CAS; Central Asian, EAS; Eastern Asian, EUR; European, FIN; Finnish, MDE; Middle Eastern, SAS; South Asian. [file MDS-40-1433-s001.jpg]

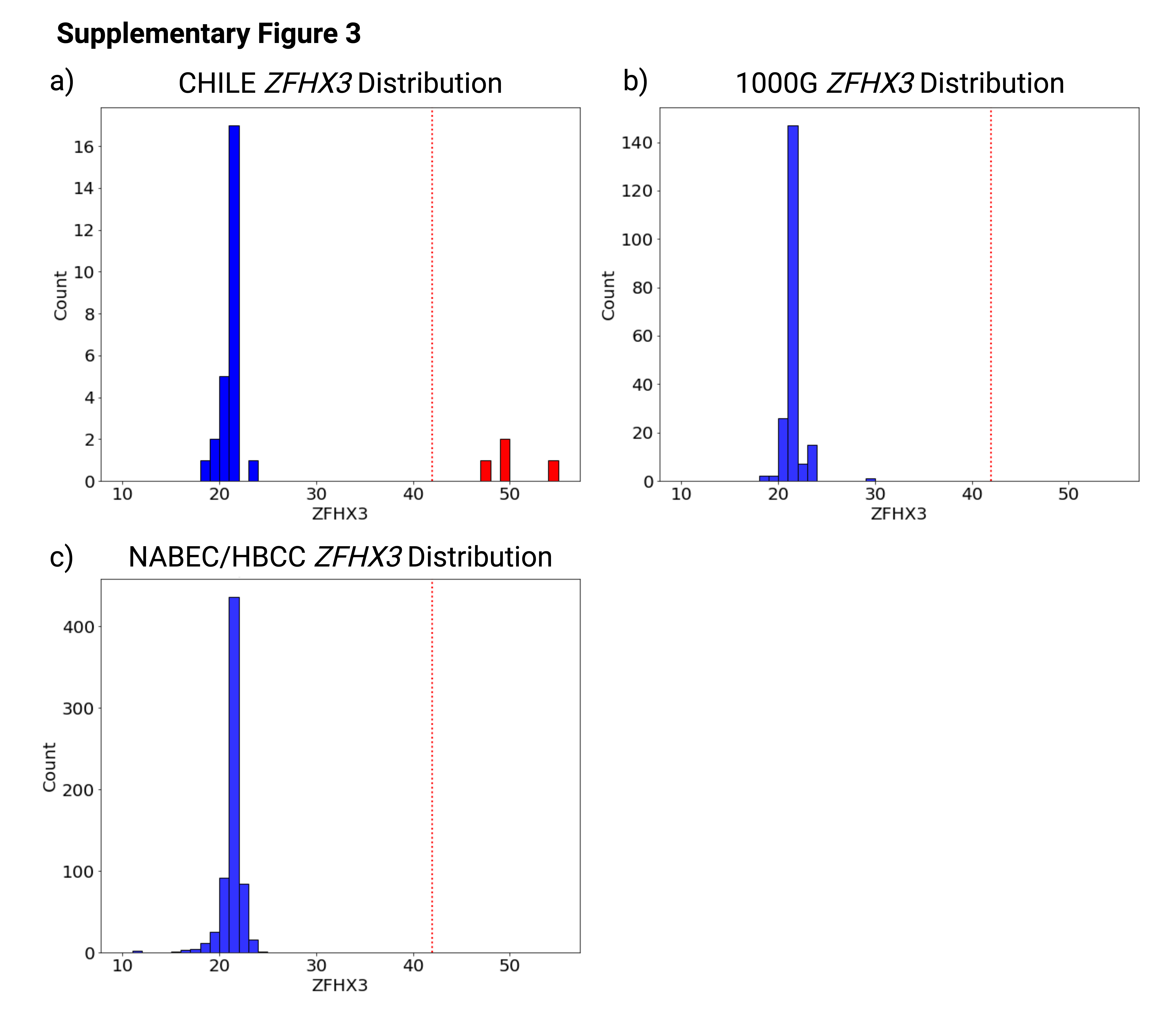

Supplement: Supplementary file 3 — Figure S3. Control Dataset Repeat Size Estimation. (a) Distribution of ZFHX3 GGC repeat lengths in the Chilean samples (n = 15), read indicates pathogenic length carrier. (b) Distribution of ZFHX3 GGC repeat lengths in the 1000G control cohort (n = 100) comprising individuals of mixed ancestry. (c) Distribution of ZFHX3 GGC repeat lengths in the NABEC/HBCC control cohort (n = 338) comprising individuals of European and African‐admixed ancestry. The red dotted line marks the pathogenic threshold. [file MDS-40-1433-s005.jpg]

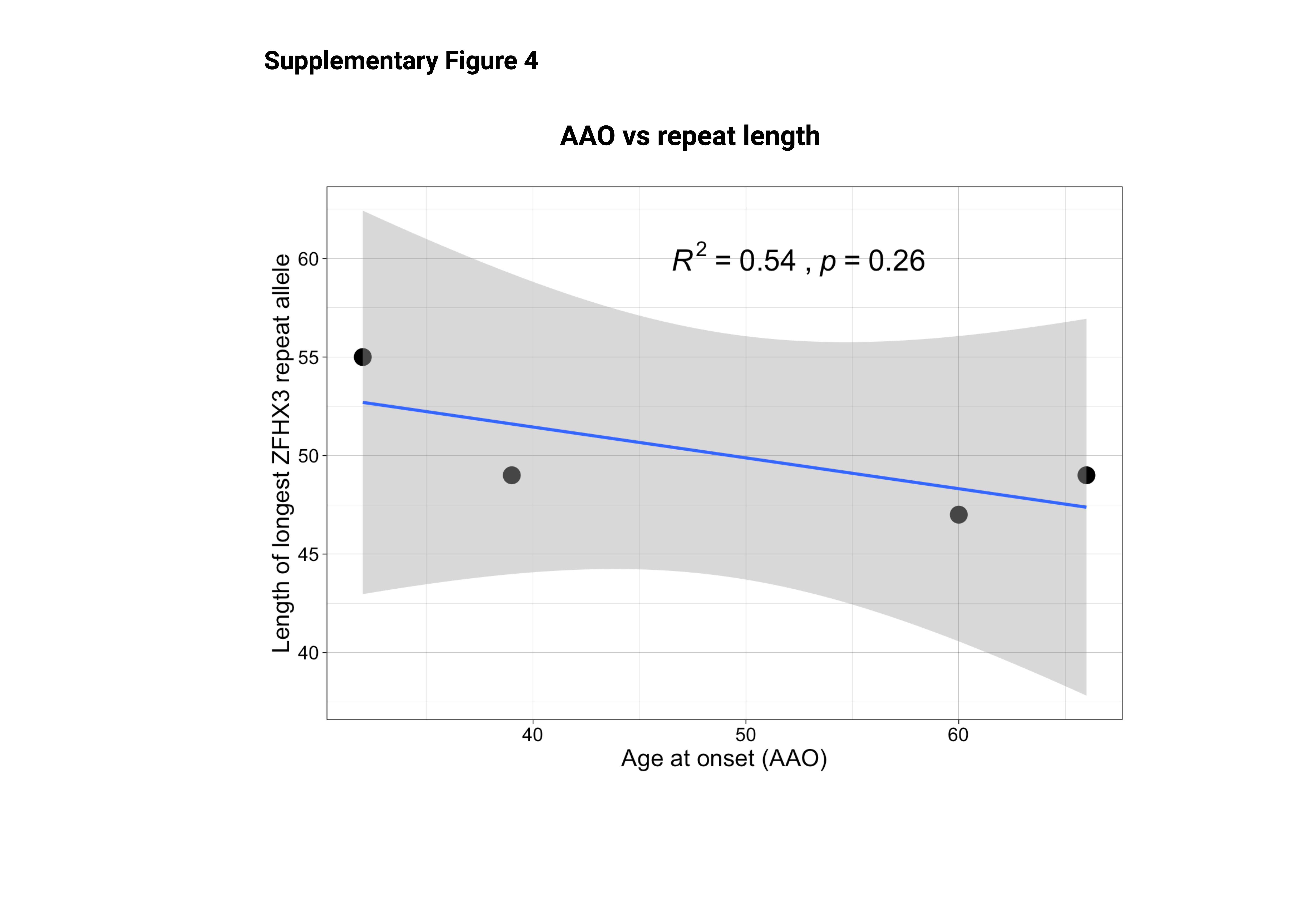

Supplement: Supplementary file 4 — Figure S4. Inverse Correlation Between ZFHX3 GGC Repeat Length and Age at Onset in SCA4 Patients. A negative trend is observed, but it is not statistically significant (R 2 = 0.54, P = 0.26). [file MDS-40-1433-s004.jpg]
